# Cytohistological Correlation of Thyroid Cases with Emphasis on Papillary Thyroid Carcinoma and Analysis of the Causes of Diagnostic Errors on Cytology

**DOI:** 10.5146/tjpath.2025.13787

**Published:** 2025-05-31

**Authors:** Soundarya Soundarya, S. Mary Theresa Sylvia, Banushree Chandrasekhar Srinivasamurthy

**Affiliations:** Department of Pathology, Indira Gandhi Medical College and Research Institute, Pondicherry, India

**Keywords:** Thyroid fine needle aspiration cytology, Cytohistology correlation, Papillary thyroid carcinoma

## Abstract

***Objective: *** Fine needle aspiration cytology is the first line of investigation for thyroid lesions. Despite standard reporting formats, the diagnostic accuracy varies across institutions. In this study, we have reviewed our discordant cases on cytology and histopathology and analyzed the diagnostic errors.

***Material and Methods:*** The thyroid cases with discrepant cytology and histopathology reports for a period of five years were analyzed for diagnostic errors. The papillary thyroid carcinoma (PTC) cases were studied in detail for all diagnostic parameters. Nuclear scoring was used to improve the detection of PTC.

***Results: *** Of the 166 cases, 18 (10%) had discrepant diagnoses. The sensitivity was 65.62% (CI 46.81-81.43%), specificity 94.78%, positive predictive value 75%, negative predictive value 92.03%, positive likelihood ratio 12.56, negative likelihood ratio 0.36, false positive rate 5.2%, false negative rate 34.3% and accuracy 89.16%.

False negative (malignant cases diagnosed as benign) was due to inadequate/wrong site sampling, benign clusters/ cyst macrophages, marginal flares, thin colloid, larger fragments of calcification, and subtle nuclear features. An interesting flower head-like structure was observed in PTC cases. Nuclear scoring on false negative cases improved our diagnostic accuracy. False positivity was due to vigorous aspiration and over-interpretation of nuclear features.

***Conclusion:*** Analysis of our discrepant cases highlighted the importance of multiple passes, sampling all nodules, and ultrasound-guided aspiration to reduce sampling error. Application of nuclear scoring reduced overdiagnosis and missing out on PTC. Tissue fragments and hypercellularity were the major misleading factors in false positive cases.

## INTRODUCTION

Fine needle aspiration cytology (FNAC) is an essential investigation of thyroid lesions to avoid unnecessary surgeries. Among the various classification systems, the Bethesda system for reporting thyroid cytology (TBSRTC) is widely followed and validated. It classifies thyroid lesions into 6 diagnostic categories: (i) non-diagnostic; (ii) benign; (iii) atypia of undetermined significance; (iv) follicular neoplasm; (v) suspicious for malignancy; and (vi) malignant (1,2). However the diagnostic accuracy of cytology varies across institutions. In the literature, false negative (malignant reported as benign on cytology) range from 10.68 to 19.7% and false positive (benign reported as malignant on cytology) range from 1.9 to 35.7 % (3–8). There are many studies on cytology and histology correlation of thyroid but only a few have reviewed the slides to identify the features that caused the misdiagnosis (6–8). In this study, we have analyzed the discrepant thyroid cases on cytology and histopathology in our institute and studied the benign features that mimic malignancy and cytological features that misled the cytopathologist on FNAC to improve our diagnostic accuracy. We have also assessed all the reported features of papillary thyroid carcinoma (PTC) and the nuclear scoring method in our discrepant cases to find if it would improve our diagnostic accuracy.

## MATERIALS and METHODS

This is a retrospective descriptive study done in the Department of Pathology of our Institute. All the thyroid histopathology cases were collected from the Department of Pathology for a period of 5 years. Fine needle aspiration cytology (FNAC) of these cases done through the palpation method, and ultrasound guided in few cases, were collected. The cytology reports and clinicopathologic details were retrieved from the archives of the department. The cases with discrepant diagnoses between cytology and histopathology were filtered. The cytology and histopathology slides of these discrepant cases were reviewed by the authors for architectural, nuclear features, atypia, and parameters that misdirected the pathologist from the accurate diagnosis. Sampling errors, suboptimal specimen preparation, and interpretation errors that caused underdiagnosis or overdiagnosis of the specimen were analyzed. The PTC cases were studied in detail. All the features documented in the literature for PTC like nuclear enlargement, moulding, grooving, micronucleoli, pale nuclei, membrane irregularity, elongation/spindling of cells, 3D/papillary fragments, and thick colloid were assessed on the cytology slides (9). In addition, the nuclear scoring system (Table 1) by Nikiforov et al. (10) originally used to diagnose Non-invasive Follicular Thyroid Neoplasm with Papillary-Like Nuclear Features (NIFTP) was applied on cytology smears to check if it improved the detection of nuclear features of PTC and reduced interobserver variability.

**Table 1:** Nuclear scoring: (1 point each) (10)

**Size and Shape**		Enlargement, Elongation, Crowding, Overlapping
**Membrane irregularity**		Irregular membrane, Grooves, Folds, Intranuclear inclusions
**Chromatin**		Clearing, Margination, Glassy, Evenly fine
**Score**	**0- **	**Absent or slightly present**
	**1-**	**Present or well developed**
**Total score**	**0 or 1**	**Non diagnostic**
	**2 or 3**	**Diagnostic of Papillary carcinoma**

Statistical analysis: Categorical variables were represented as percentages and numbers. The results were reported as False negative rate, False positive rate, sensitivity, specificity, Positive predictive value, Negative predictive value, and accuracy.

All procedures performed in the current study were approved by the Institute research and ethics committee (reference number IRC 202283 10.10.2022 , No 441/IEC-36/PP2 01.12.2022) and were in accordance with the revised 2024 Helsinki Declaration and its later amendments. Formal written informed consent was not required with a waiver by the appropriate Institute’s research ethics committee.

## RESULTS

The total number of histopathology resected thyroid specimens received during the study period of five years was 166 cases. The age range was 3 to 71 years. Males made up 5% (9 cases) and females 94% (157 cases). Among the total, 32 (19%) cases were malignant; 134 (80%) cases were benign on histopathology. The characteristics of the 166 cases are summarized in Table 2. Of the 166 cases, 18 thyroid cases (10%) had discrepant cytology and histopathology diagnosis. Among the 18 discrepant thyroid cases, eleven cases were false negative (61%) (malignant on histopathology but reported as benign on FNAC) and seven cases were false positive (38%) (benign on Histopathology but reported as malignant/atypia/suspicious on FNAC). The sensitivity was 65.62% (CI 46.81-81.43%), specificity 94.78% (CI 89.53-97.87%), positive predictive value 75% (CI 58.30-86.55%), negative predictive value 92.03% (CI 87.72-94.91%), positive likelihood ratio 12.56 (CI 5.85-26.96%), negative likelihood ratio 0.36 (CI 0.22-0.59%), false positive rate 5.2%, false negative rate 34.3% and accuracy 89.16% (CI 83.40- 93.45%).

**Table 2:** Characteristics of the 166 thyroid cases

**Characteristics**	**No. of Cases**
Age range	3-71 years
Males	9 (5%)
Females	157 (94%)
Thyroglossal cyst	4 (2%)
Colloid nodule	8 (4%)
Thyroid follicular nodular disease	103 (62%)
Papillary thyroid carcinoma	29 (17%)
Invasive encapsulated Follicular variant of papillary thyroid carcinoma (IEFVPTC)	2 (1%)
Follicular thyroid adenoma	19 (11%)
Follicular carcinoma	1 (0.6%)

### False Negative Cases

Out of 18 mismatched diagnoses, eleven cases were reported as benign in FNAC and were malignant on histopathology. The FNAC diagnosis in false negative cases were eight (72%) thyroid follicular nodular disease with cystic changes (Category II), one (9%) inadequate sampling (category I), one (9%) colloid goiter with cystic change, and one case (9%) repeated twice with FNAC categorization as Category III (first FNAC) and II (Repeat FNAC). On histopathological examination, eight of these cases were PTC-classical subtypes, two invasive encapsulated follicular variants of papillary carcinoma (IEFVPTC), and one PTC (1mm size) classical subtype with colloid goiter. We did not receive any cases of non-invasive follicular thyroid neoplasm with Papillary-Like Nuclear Features (NIFTP). Table 3 summarizes the false negative cases.

**Table 3:** False negative cases (Malignant cases missed on cytology)

**Cytology diagnosis (No of cases)**	**Histopathology diagnosis**
Category 1- Colloid nodule (1 case)	Thyroid follicular nodular disease with papillary carcinoma classic subtype (1 mm)
Category 2 Nodular goiter with cystic change (8 cases)	Papillary carcinoma-classic subtype (6)/ Invasive Encapsulated follicular variant of papillary carcinoma (2)
Category I Inadequate (1 case)	Papillary carcinoma-classic subtype
Atypia of undetermined significance-1st FNAC and Nodular colloid goiter-2nd FNAC (1)	Thyroid follicular nodular disease with one nodule showing papillary carcinoma classic subtype

**Figure 1:**
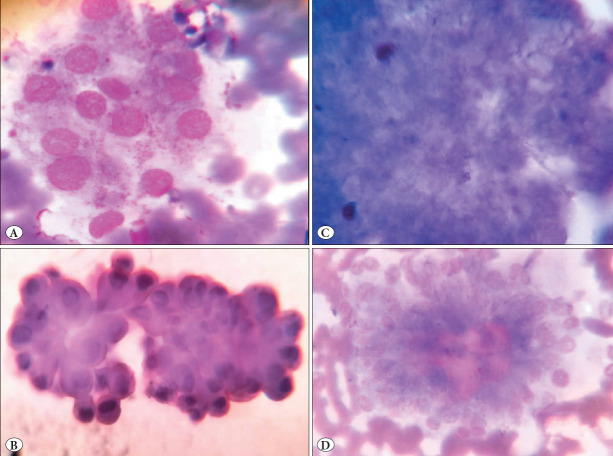
Thyroid follicular nodular disease cases diagnosed as Papillary thyroid carcinoma (False positive cases) - Mimics malignancy **A)** Tissue fragments (H &E 100X). **B)** Hypercellularity (H & E 100X). **C)** Nuclear overlapping and overcrowding. (Leishman 400X), **D)** Swirl like structures. (Leishman 400X). Inset shows intranuclear pseudoinclusions. (PAP 400X).

On review of the false negative slides, inadequate sampling and wrong site sampling were observed in the case of colloid goiter with PTC (1mm size) classical subtype. Eight cases reported as thyroid follicular nodular disease with cystic change had a significant number of tissue clusters caught within blood clots masking the nuclear features of PTC. The abundance of benign features like thin colloid, benign follicular clusters, cyst macrophages, marginal flares (Figure 1), and large fragments of calcification (Figure 1) misled the pathologist. One case had inadequate material and suboptimal staining. One case repeated twice was a thyroid follicular nodular disease with one of the nodules showing PTC. On review, the first aspirate from the malignant nodule had nuclear features of PTC and the second aspirate from the adjacent nodule showed thyroid follicular nodular disease.

On analyzing the nuclear features so far reported in the literature (9) like nuclear enlargement, moulding, grooving, micronucleoli, pale nuclei, membrane irregularity, elongation/spindling of cells, 3D/papillary fragments, and thick colloid in PTC cases (11 cases), nuclear enlargement, nuclear grooving, and pale nuclei were noted in most of the misinterpreted cases. Nuclear moulding, papillary fragments, and membrane irregularity were absent in all the cases. Nuclear inclusions, micronucleoli, and thick colloid were seen in one case each (9%). Elongation was present in four cases (36%). A very small fragment of a papilla was seen in one case (9%) (Figure 1). The IEFVPTC case showed only subtle nuclear features of PTC.

The nuclear scoring system (10) was applied to the eleven cases of PTC. Six of the eleven cases (54%) of PTC reached a significant score of 2/3. Of the parameters of the nuclear scoring system, nuclear enlargement, grooves, and pale chromatin were seen in all eleven cases. Papillary fragments, membrane irregularity, nuclear moulding, psammoma bodies, and swirls were absent in all the cases. An interesting observation of two-dimensional clusters with two or more rows of nuclei arranged like a flower head-like pattern around the central colloid was present in all the cases (Figure 1). The IEFVPTC case showed an insignificant nuclear score of 1.

### False Positive Cases

Out of 18 mismatched diagnoses, seven cases reported as malignant in FNAC were thyroid follicular nodular disease on histopathology. Cytology reports were two cases (28.5%) of oncocytic neoplasm, one case (14.2%) suspicious for PTC (category V), 2 cases (28.5%) of PTC (Category VI), one case (14.2%) of follicular neoplasm (Category IV), and one case (14.2%) of category III atypia of undetermined significance.

**Figure 2:**
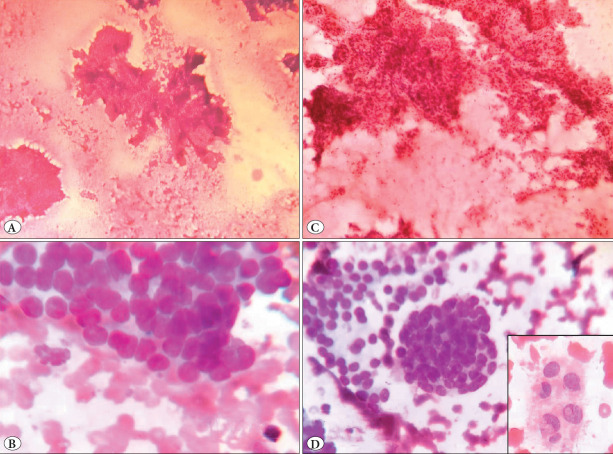
Papillary thyroid carcinoma cases diagnosed as benign (False negative). **A)** Benign clusters with marginal flares. (Leishman 400X), **B)** Small fragment of a papilla. (PAP 400X), **C)** Large fragment of calcification (Leishman 400X), **D)** Flower head like arrangement of follicular cells (Leishman 400X).

On review of the slides, the parameters that led to the diagnosis of malignancy were a highly cellular aspirate (all seven) (Figure 2), micro biopsy fragments (three cases, 42%) (Figure 2) causing overlapping, overcrowding, (Figure 2), and swirls (Figure 2), crushing of the cells leading to artefactual elongation of cells (one case), drying artifacts on Leishman stain (two cases), Tissue clusters caught within blood clot (three cases, 42%) resembling papillary fragments, and reactive fibroblasts (two cases) were mistaken as abnormal nuclear features. Nuclear grooves and intranuclear pseudo inclusions (Figure 2 inset) were seen in two cases (28%).

A nuclear scoring system (10) was applied to the three cases suspected of PTC on cytology. None of them had significant scores.

## DISCUSSION

Standard reporting formats like the TBSRTC have improved the categorization of thyroid lesions on FNAC and improved diagnostic accuracy (11). However, the diagnostic accuracy of thyroid lesions using the TBSRTC on FNAC varies across institutions, ranging from 63.9% to 97% (3–8). Sharma, Silva et al., Syed et al., and Osseis et al. (5–8) have conducted similar studies comparing the cytology and histopathology diagnosis of thyroid lesions. However, these studies have not analyzed the factors that caused the misdiagnosis. We conducted this study to evaluate the diagnostic accuracy of thyroid cytology at our Institute and reviewed the slides to identify the causes for the diagnostic errors and thereby improve the diagnostic accuracy of our Institute.

The sensitivity of 65.62% (CI 46.81-81.43%) of our study is lower compared to the studies by Sharma, Silva et al., Syed et al., and Osseis et al. (82.3-94.4%) (5–8). Our sample size (166 cases) being lower than all these studies may be the limiting factor. The specificity (94.78%) is higher than Silva et al., Syed et al., and Osseis et al. and lower than Sharma (98%). Diagnostic accuracy (89.16%) of our study is lower than Sharma (97%) and higher than Silva et al. (87.9%). The false positive rate (5.2%), of our Institute is higher compared to the study by Sharma (1.9%) and lower than the studies by Osseis et al. (51.56%) and Syed et al. (35.7%) (5,7,8). The false negative rate (34.3%) of our study is higher than the rate reported by all the above authors (10.5-17.6%). Table 4 compares the sensitivity, specificity, positive predictive value, negative predictive value, and accuracy of our study with studies in the population of the same country and across the world.

**Table 4:** Comparison of results of our study of cytology and histopathology correlation of thyroid cases with similar studies

**Statistics**	**Results of our study**	**Sharma **(5)	**Silva et al. **(6)	**Syed et al. **(7)	**Osseis et al. **(8)
Sensitivity	65.62%	89.5%	94.4%	82.3%	89.31%
Specificity	94.78%	98%	86.9%	64.3%	48.44%
PPV	75%	84.6%	86.7%	73.6%	78%
NPV	92.03%	98.6%	87.9%	75%	68.89%
Accuracy	89.16%	97%	87%	63.9%	75.89%
FPR	5.2%	1.9%	13.2%	35.7%	51.56%
FNR	34.3%	10.5%	12%	17.6%	10.68%

The false negative rate (malignant cases reported as benign) of our institute was 34.3%. This is very high compared to similar studies (5–8). It is important to improve our detection of malignant cases since it will delay the treatment of malignancy and may lead to upstaging of the tumor. On review of these slides, the cause in two cases was sampling error (presence of small focus <1mm (previously classified as microcarcinoma)/one of the nodules in thyroid follicular nodular disease showing malignancy). Many authors have observed similar detection of small carcinomas on histopathology. Negro et al. reported the incidental detection of thyroid carcinoma in 8.6% of resected specimens of thyroid follicular nodular disease (12). Abou-Foul et al. reported 19.4% of cases reported as benign on FNAC to have malignancy on cytology (13). Zhu et al. had also observed sampling error as the major reason for false negative cases and suggested multiple passes to reduce the sampling error (14). Sharma reported cystic PTC and papillary microcarcinoma as the major reasons for false negative cases (5). Hence sampling all nodules, multiple passes, and ultrasound-guided sampling in cases of PTC occupying a microscopic focus would have helped us to minimize the error. The European Thyroid Association guidelines recommend ultrasound-guided FNAC of all thyroid nodules (15). Training the cytopathologist on the basics of thyroid ultrasound would help to implement this method in hospitals with less manpower. Kuzan et al. compared the adequacy of thyroid cytology material with the number of passes and concluded that two passes yielded better material (16). We observed that multiple passes translating to sampling different nodules would help to improve diagnosis as we found one of the nodules in a thyroid follicular nodular disease to be malignant.

The presence of abundant benign features like plenty of thin colloid, benign clusters of follicular cells, macrophages, and large fragments of calcification (Figure 1) were important misleading factors in eight of the cases reported as thyroid follicular nodular disease which were PTC on histopathology. Monappa and Kudva observed similar misleading factors of a mixture of benign clusters causing diagnostic errors (17). Being aware of the possibility of a mixture of benign clusters and assessing each pass separately with the location/nodule would help to minimize this error.

We applied the nuclear scoring by Nikiforov et al. (10) originally used by him to diagnose NIFTP on histopathology slides as a method to identify the papillary nuclear features. The application of the score on false negative cytology smears increased the detection rate of PTC. Six of the eleven (54%) PTC cases had significant scores. Applying a standard scoring reduced our subjective variation in the interpretation of nuclear features. In contrast, IEFVPTC cases had only subtle features with insignificant nuclear score <1. The literature also documents subtle nuclear features in IEFVPTC difficult to appreciate in cytology similar to our findings in two cases of IEFVPTC (16). This highlights the limitation of cytology in the diagnosis of grey zone lesions where molecular studies would be necessary on the cytology material.

Kumari et al. (9) have studied the cytology of histologically confirmed PTC cases for nuclear features of PTC and additional parameters like giant cells, macrophages, cellular swirls, and psammoma bodies. Their study concluded that the presence of five or more cytological features (papillae with cores, cellular swirls, nuclear grooves, intranuclear inclusions, and psammoma bodies) improved the positive predictive value to 78.95% and negative predictive value to 83.33% (9). In contrast, papillary fragments, membrane irregularity, nuclear moulding, psammoma bodies, and swirls were absent in the PTC cases of our study. The development of a standardized scoring system for PTC and validation would improve the detection of cytology smears.

In addition, we observed two-dimensional clusters with two or more rows of cells arranged around the central colloid as flower-head-like structures in six of the eleven cases of PTC on the re-evaluation of the false negative cases. Sen et al. reported rosette-like structures on thyroid cytology in columnar cell variant of PTC (18). In contrast, Aquila et al. studied the presence of rosettes on thyroid cytology smears and did not find any true rosettes on PTC or benign slides They have reported pseudorosettes in 33 cases of PTC variants (19). The authors have classified the follicular cell arrangement into rosettes, microfollicles, and papillae. The structure we observed had more than two rows of cells in contrast to the microfollicles and did not have merging cytoplasmic processes as in true rosettes described by Aquila et al (19). In the absence of true papillae presence of such two-dimensional clusters might give a clue for further analysis. However, we have to study the parameter in a larger number of cases to validate the finding.

The false positive rate (benign cases reported as malignant) of our Institute was 5.2%. This is higher than the rate reported by Sharma (1.9%) and lower than Silva et al. (12%), Syed et al. (35.6%), and Osseis et al. (51.56%) (5–8). These cases in our study were misinterpreted mainly due to the high cellularity and tissue/stromal fragments. These microbiopsies were due to the vigorous aspiration technique used and caused overlapping and overcrowding of cells. These fragments have been reported in FNAC of Grave’s disease and adenomatous hyperplastic nodules of the thyroid (20). Similar pitfalls were observed by Jing and Michael, and Faser et al. (20,21). The papillary-like tissue/stromal fragments had different morphology like folded sheets, with fibro-collagenous tissue in between and were entrapped in blood. Stromal fragments have not been extensively studied or included in the criteria in thyroid cytology. We found only one study by Mai and Hogan which classified the stromal fragments into types 1 and 2 based on the length, collagen content, and entrapped blood and follicular cells. But they also observed overlapping features (22). Vigorous aspiration technique should be ruled out during the evaluation of slides with excessive tissue fragments and cellularity before the diagnosis of malignancy.

Nuclear features like nuclear grooves and intranuclear inclusions have been overdiagnosed in highly cellular smears in our false positive cases. In cases of suspicion of PTC, the presence of nuclear grooves, rare intranuclear inclusions, and reactive fibroblasts caused the interpretation errors. Suboptimal smears with drying artifacts and crushing mimicked nuclear elongation and enlargement. Sharma and Zhu et al. also reported the overdiagnosis of nuclear features of PTC to be the reason for false positivity (5,14). These features can also be seen in benign lesions like chronic lymphocytic thyroiditis, thyroid follicular nodular disease, and hyalinizing trabecular tumors (23). Bhat et al. reported increased sensitivity, and specificity of 90.5% and 100% by scoring the percentage of nuclear grooves. Nonneoplastic lesions showed <10% and PTC ≥20% (24). Prabhu and Umashankar et al. had also proposed scoring criteria to reduce false positive PTC (25). On application of the nuclear scoring, none of these cases reached a significant score. Hence a validated combined scoring system for nuclear features would help to minimize false positive and negative rates as discussed previously. It would decrease subjective errors and avoid missing and overdiagnosis of nuclear features. Incorporation of such scoring in the standard reporting formats will help to prevent pitfalls in the diagnosis.

## CONCLUSION

Analysis of our discrepant cytology cases helped us to understand the factors that led to the diagnostic error. It also highlighted a few corrective measures that we can practice. Multiple passes, sampling all nodules, and ultrasound-guided aspiration will help us to reduce sampling error. Application of nuclear scoring would help to reduce overdiagnosis and missing of nuclear features in PTC. Vigorous aspiration techniques should be considered while evaluating smears with multiple tissue fragments and hypercellularity. The presence of benign clusters should not mislead the diagnosis of malignancy. Assessing each pass separately with the location/nodule/ imaging findings would help to reduce errors. The presence of subtle features like flower head-like structures and small fragments of a papilla should be observed in all cases.

## Conflict of Interest

The authors have no conflicts of interest to declare.
